# Efficacy of Tooth Bleaching With Prior Application of Two Different Desensitizing Agents: An In Vitro Study

**DOI:** 10.7759/cureus.41361

**Published:** 2023-07-04

**Authors:** Revathy Parthasarathy, Senthilkumar Kumarappan, Sankar Vishwanath, Yashini Thanikachalam, Srividhya Srinivasan, Shwetha Ramachandran

**Affiliations:** 1 Conservative Dentistry and Endodontics, Sree Balaji Dental College and Hospital, Chennai, IND; 2 Conservative Dentistry and Endodontics, Private Practitioner, Chennai, IND; 3 Conservative Dentistry and Endodontics, KSR Institute of Dental Science and Research, Tiruchengode, IND; 4 Conservative Dentistry and Endodontics, Chettinad Dental College and Research Institute, Chennai, IND; 5 Conservative Dentistry and Endodontics, Sri Venkateswara Dental College and Hospital, Chennai, IND

**Keywords:** tooth bleaching, sodium fluoride, potassium nitrate, digital spectrophotometer, carbamide peroxide, bleaching efficacy

## Abstract

Objective

To compare and evaluate the efficacy of tooth bleaching with prior application of two different desensitizing agents such as sodium fluoride and 5% potassium nitrate.

Materials and methods

A total of 108 extracted human maxillary central incisors were stained in black coffee solution and stored in artificial saliva for colour stabilization. The specimens were randomly divided into three groups (n = 36) according to the following protocols: (a) bleaching without desensitizer, (b) bleaching with prior application of sodium fluoride, and (c) bleaching with prior application of 5% potassium nitrate. After fabricating customized trays, desensitizers were applied for 10 minutes followed by 16% carbamide peroxide bleaching gel, which was in contact with the teeth for three hours. The bleaching efficacy was evaluated at baseline (after staining), 3rd day, 7th day, and 14th day using a digital spectrophotometer.

Results

There was an increase in the overall colour change (∆E) from baseline to 14th day, which was statistically significant at cervical, middle, and incisal thirds of the teeth between the three groups with the sodium fluoride group showing decreased ∆E.

Conclusions

Carbamide peroxide (16%) showed improved whitening efficacy from baseline to the 14th day with increasing median values at all time periods. The sodium fluoride group showed decreased ∆E value when compared to other groups.

## Introduction

Over the past three decades in dentistry, patients' priority has moved towards aesthetics or the aesthetic appearance of the teeth. The increase in the demand for whiter teeth has paved the path for various teeth whitening procedures. The colour of an object is influenced by the source of the light, the object which is seen, and the person seeing the object [1]. The colour of the teeth is mainly produced by the dispersion of light [2].

Mimicking the natural tooth colour is one of the crucial steps during tooth bleaching. Tooth whitening or bleaching is considered one of the most common, non-invasive techniques that offer the ideal whitening of teeth. Bleaching of vital teeth can be done by in-office and at-home bleaching techniques. Since its introduction, carbamide peroxide (CP) (10-20%) is the most used at-home bleaching agent, which is worn during nighttime for at least two to four weeks for whitening the teeth [3]. According to Llena et al., 16% CP was an efficient, stable, and safe tooth-brightening procedure, over a long period of time [4].

Tooth colour measurement is done by various methods ranging from subjective visual comparison to objective electronic shade measurement instruments, such as colourimeter, spectrophotometer, and digital image analyzer [5]. Spectrophotometers work by measuring the amount of reflected light in a visible spectrum for each wavelength and are said to have better efficacy and working span than colourimeters.

One of the most common adverse effects of vital bleaching treatment is post-bleaching sensitivity, which is seen among 43-80% of the patients [6]. The higher incidence of post-bleaching sensitivity is due to the penetration of peroxide molecules into the pulp chamber leading to transient inflammation of the pulp. To overcome this adverse effect, desensitizing agents were applied on the teeth [7,8].

The application of desensitizing agents has been an active approach in treating post-bleaching sensitivity. The mechanism of action of a desensitizing agent either blocks the dentinal tubules or desensitizes the nerve [1,9]. Some of the desensitizing agents available are potassium nitrate (KNO3), sodium fluoride (NaF), stannous fluoride, sodium monofluorophosphate, and strontium fluoride.

As these desensitizing agents have different mechanisms of action, in the present study, the effect of NaF and 5% KNO3 on the bleaching efficacy of 16% CP was evaluated on the cervical, middle, and incisal thirds of the teeth.

## Materials and methods

The present study protocol was reviewed and approved by the Institutional Human Ethics Committee of Chettinad Academy of Research and Education (CARE; approval number: 196/IHEC/1-19). Informed consent was obtained from the patients whose tooth specimens were obtained. A total of 108 extracted human maxillary central incisors were used in this study. The extracted teeth were stored in distilled water until use. Teeth with caries, fractures, any stains or discolouration, developmental anomalies, attrition, and existing restoration were excluded from the study.

All the teeth were stained using a black coffee solution. The black coffee solution was prepared by adding 5 g of coffee powder (Nescafé Sunrise, Nestle India Ltd., New Delhi, India) to 100 ml of distilled water and boiling for five minutes. The specimens were immersed in the staining solution for seven days, which was replaced every 24 hours with a freshly prepared solution. The staining solution was in contact with the specimens throughout the staining procedure. After seven days of staining, the specimens were stored in artificial saliva, which was changed each day, for two weeks for colour stabilization [8]. Before the baseline spectrophotometric reading, the black coffee sediments or impurities that were formed on the teeth specimens were removed using a rubber cup with a mixture of saline and pumice powder at low speed for 30 seconds.

After two weeks of colour stabilization, the specimens were randomly allocated to three experimental groups as follows (n = 36): Group 1: bleaching without desensitizer; Group 2: application of NaF before bleaching; Group 3: application of 5% KNO3 before bleaching.

**Figure 1:**
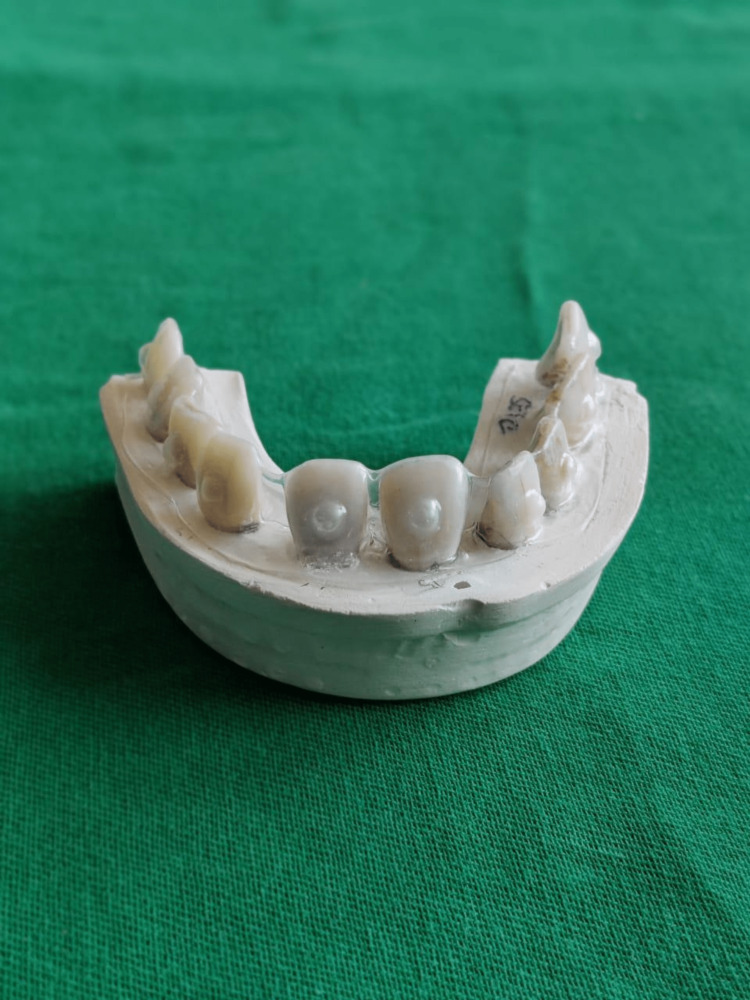
Specimens with the customized bleaching tray

All the specimens were mounted on the plaster of Paris in an arch form, according to group specification, and bleaching trays were fabricated by the vacuum-formed process using soft-vinyl material of 3 mm thickness (Figure 1). Before the tray fabrication, a reservoir of approximately 2 mm thickness was formed using resin composite, to provide space for the bleaching gel.

**Figure 2:**
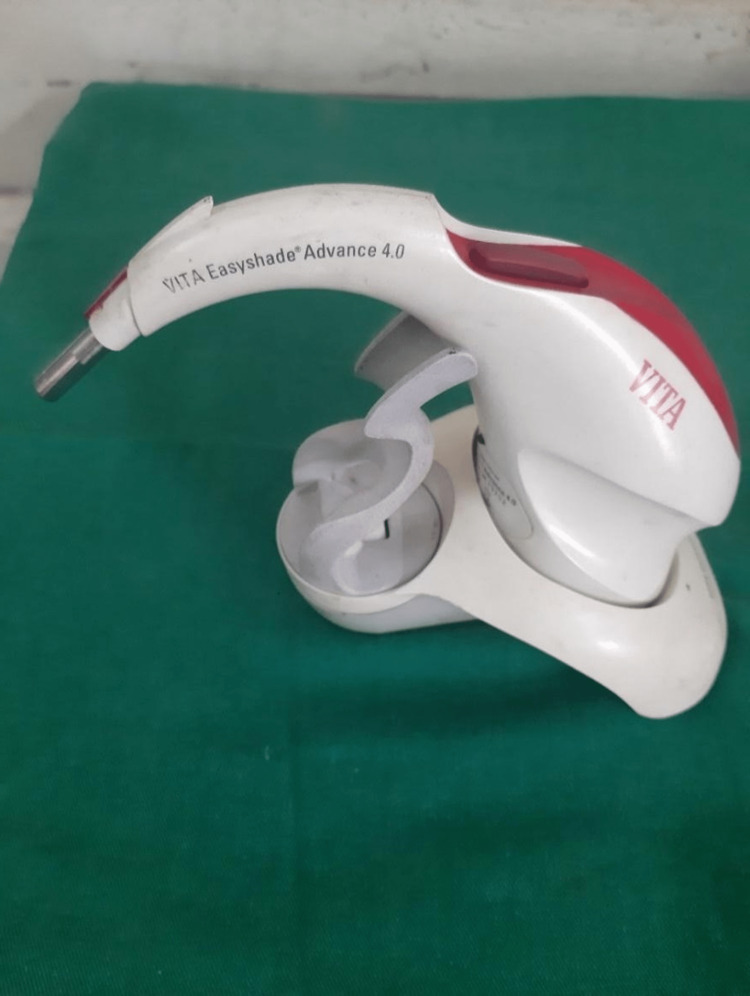
Digital spectrophotometer (VITA Easyshade Advance 4.0 spectrophotometer)

After the staining procedure, the colour measurement was done using a digital spectrophotometer (VITA Easyshade® Advance 4.0, VITA Zahnfabrik, Bad Säckingen, Germany) (Figure 2) before bleaching, which acts as the baseline value. Colour measurement was done on the cervical, middle, and incisal thirds of the teeth, following which the specimens were washed thoroughly under tap water and dried using absorbing paper.

In Group 1, 16% CP (Pola Night, SDI Limited, Itasca, IL) was applied on the customized bleaching tray, which was fitted to the cast containing the specimen.

In Group 2, NaF (Fluocal Gel, Septodont Healthcare India Pvt. Ltd., Raigad, India) was applied for 10 minutes to an approximate thickness of 1 mm. The desensitizer was then removed using cotton-tipped rods and washed thoroughly under tap water. Following this, the bleaching gel (16% CP) was applied on the customized bleaching tray, which was then, fitted to the teeth.

In Group 3, 5% KNO3 (Senquel Foaming Medicaled Oral Gel, Dr. Reddy's Laboratories, Hyderabad, India) was applied to approximately 1 mm and allowed for 10 minutes. It was later removed using cotton-tipped rods and washed under tap water. The bleaching gel (16% CP) was applied to the customized bleaching tray, which was then fitted to the teeth.

In all the groups, the bleaching agent was in contact with the teeth for three hours and this procedure was repeated for 14 days. At the end of each bleaching session, the specimens were washed, dried, and stored in artificial saliva at room temperature to rehydrate the teeth before colour measurements.

**Figure 3:**
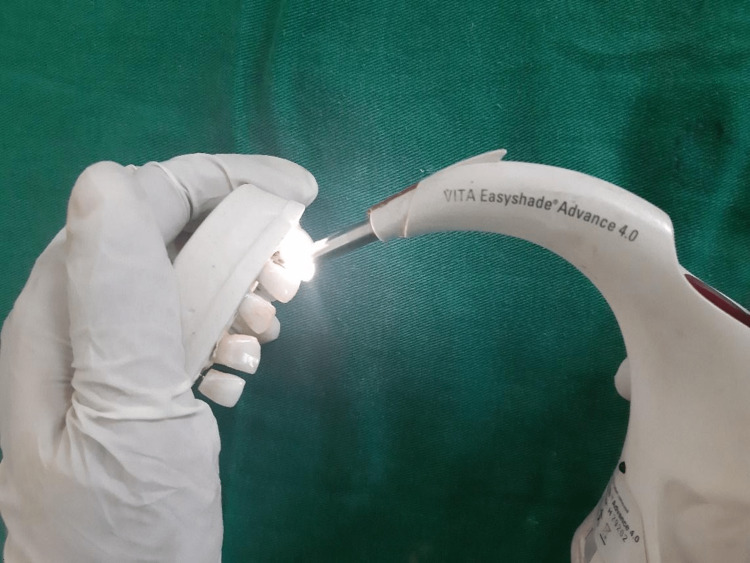
Colour measurement of the specimen was done using a digital spectrophotometer

Following 24 hours in artificial saliva, the specimens were dried using absorbing paper and colour measurements were taken using a digital spectrophotometer (Figure 3).

The colour measurements were taken on the 3rd day, 7th day, and 14th day of bleaching in the afternoon with sunlight and room illumination [10]. The digital shade measurement was done on the cervical, middle, and incisal thirds of the standardized area of the crown structure. The overall colour change was symbolized as ΔE by a digital spectrophotometer, which was from the Commission Internationale de l'Eclairage (CIE). The following equation was used: ΔE = √ (L ∗2− L ∗1)2 + (a ∗2− a ∗1)2 + (b ∗2− b ∗1 2 )2, where L* stands for the degree of lightness, a* stands for the presence of red pigments (positive), negative a* is for green pigments, b* positive is for yellow pigments, and negative b* is for blue.

## Results

There was a statistically significant increase in the overall colour change ∆E from baseline to the 14th day at the cervical, middle, and incisal thirds of the teeth in all three experimental groups (Table 1). It can also be noted that in all the thirds, the NaF group showed decreased numerical values when compared to the control and KNO3 groups.

**Table 1:** Distribution of samples based on the mean rank score at the cervical, middle, and incisal thirds at repeated measurements * Statistically significant. NaF: sodium fluoride; KNO3: potassium nitrate.

Groups	Baseline			3^rd^ day			7^th^ day				14^th^ day			p-value
	Control	NaF	KNO_3_	Control	NaF	KNO_3_	Control	NaF	KNO_3_	Control		NaF	KNO_3_	
Cervical	1.00	1.00	1.00	2.05	2.03	2.08	3.01	2.97	3.04	3.96		3.92	3.97	.000*
Middle	1.03	1.02	1.01	2.02	1.97	2.03	3.06	3.06	3.10	3.79		3.88	3.92	.000*
Incisal	1	1.01	1	2	2	2.04	3.00	3.01	3.03	3.97		3.96	4	.000*

The inter-group and between-group analysis in the cervical third (Table 2) did not show any statistically significant difference among the groups, i.e., all the groups performed statistically similarly on the 3rd, 7th, and 14th days. But in the middle third (Table 3), statistically significant differences between the groups were seen on the 3rd, 7th, and 14th days and only on the 14th day in the incisal third (Table 4).

**Table 2:** Kruskal-Wallis test for between-group analysis at the cervical third

	p-value			
	Baseline	3^rd^ day	7^th^ day	14^th^ day
Control	0.048	0.296	0.259	0.125
Sodium fluoride				
Potassium nitrate				

**Table 3:** Kruskal-Wallis test for between-group analysis at the middle third * Statistically significant difference seen between the groups.

	p-value			
	Baseline	3^rd^ day	7^th^ day	14^th^ day
Control	0.279	0.040^*^	0.001^*^	0.018^*^
Sodium fluoride				
Potassium nitrate				

**Table 4:** Kruskal-Wallis test for between-group analysis at the incisal third * Statistically significant difference seen between groups.

	p-value			
	Baseline	3^rd^ day	7^th^ day	14^th^ day
Control	0.007	0.172	0.081	0.038^*^
Sodium fluoride				
Potassium nitrate				

## Discussion

The colour of the teeth is affected by the thickness of dentin and enamel as it affects the transmission of light. According to O’Brien et al., a statistically significant colour difference was found among the three different regions of the teeth, which was also clinically evident [11]. Hence, in the present study, the effect of two different desensitizing agents on the colour-changing efficacy of bleaching agents was evaluated on the cervical, middle, and incisal regions of the teeth.

In the present study, 16% of CP was applied for three hours daily for 14 days. Authors have recommended the use of 15-16% CP for 14 to 21 days at a period of one to eight hours a day [12,13]. Though bleaching treatments are done between two to 28 days, many studies have most commonly used 14 days as the period [14]. Previous literature states that CP produced a better whitening effect than hydrogen peroxide over a shorter span of time [15].

According to Luque-Martinez et al., tray-delivered CP produced effective whitening slightly better than hydrogen peroxide when the overall colour change was measured with a spectrophotometer [16]. Also, the trays fabricated in soft vinyl material produced less gingival irritation than the thick, rigid material [17]. Thus, in the present study, customized trays were fabricated with soft vinyl for all the teeth, according to group specifications.

Being a flexible, accurate, and reliable instrument for colour measurement [18], a spectrophotometer was used to evaluate the overall colour change in this study. Spectrometric values showed a gradual increase in the ∆E values in the cervical, middle, and incisal thirds from baseline to the 14th day, signifying the effect of 16% CP on the tooth structure.

One of the most common adverse effects of bleaching is tooth-whitening-induced sensitivity or post-bleaching sensitivity. This sensitivity after bleaching is based on Brannstrom’s hydrodynamic theory, in which fluids inside the dentinal tubules are under constant movement when exposed to various thermal and osmotic changes. During these changes, the dentinal fluids tend to rapidly flow outward causing changes in the pressure throughout the dentin. This in turn sensitizes the nerve resulting in pain [19].

The mechanism of the bleaching agent relies on the penetration of reactive oxygen molecules and free radicles into the dentinal tubules decomposing the stain pigments. NaF, being the most common desensitizing agent used, has the mechanism of blocking the dentinal tubules by deposition of calcium fluoride crystals. This raises concern about the hindrance of these crystals on the penetration of bleaching molecules. A previous study suggests that calcium fluoride deposition might adversely affect the penetration of peroxide and the efficacy of the bleaching agents. However, due to the lack of literature regarding this problem, the use of sodium fluoride continues to be used as desensitizing agent [20].

Though various studies have concluded that NaF does not affect the efficacy of bleaching [21-23], in the present study, the results revealed that the NaF group had decreased ∆E values when compared to other groups. This is consistent with a study by Malekipour et al. [24] in which the NaF group showed decreased ΔE values when compared to other groups, proving the discolouring nature of NaF. Also, Kim et al., have described that NaF can produce discolouration when used after bleaching [25].

In the current study, the desensitizing agents applied prior to bleaching were washed in running water after 10 minutes of application. Though sodium fluoride was washed off, the calcium fluoride precipitates formed on the teeth tend to remain highly stable. In the oral environment, due to the interaction between the calcium fluoride globules with phosphate, the calcium fluoride deposits remain undisturbed. It was said that these precipitates dissolve only at an acidic pH of 5.0. This finding proves that the application of sodium fluoride, in turn, forms stable calcium fluoride deposits that are firmly adhering to the tooth structure; thus, arising concern about peroxide penetration [20]. Though the study by Kyaw et al. [26] supports the use of NaF, it was mentioned that the use of NaF ahead of bleaching was refrained by the manufacturer, and the reason behind this was not explained. Also, a recent systematic review regarding the colour-changing effect of desensitizing agents, when applied before bleaching, stated inconclusive results [27].

The limitation of this study is that it was conducted on extracted teeth, which did not mimic the natural teeth’s colour and dynamics. The effect of saliva on the remineralization of bleached teeth was not reproduced by artificial saliva.

The significance of the current study was that it compared the two commonly used desensitizing agents, which are available over the counter. Though CP improved the colour of the teeth from baseline to the 14th day, the NaF group showed decreased colour change when compared to KNO3. Despite concerns being raised earlier in the literature, the lack of evidence to support this concern had shadowed the issue and no further studies were done. Through the present study, concern has again been raised and needs further studies on similar topics.

## Conclusions

Within the limitations of this study, it can be concluded that 16% CP showed improved whitening efficacy from baseline to the 14th day at the cervical, middle, and incisal thirds of the teeth. Though CP improved the colour of the teeth, the NaF group showed decreased ΔE values than the KNO3 group, raising concern about its use as a desensitizing agent prior to bleaching. Further studies are required to assess the effect of sodium fluoride on teeth structure and bleaching.
